# Prednisolone-induced differential gene expression in mouse liver carrying wild type or a dimerization-defective glucocorticoid receptor

**DOI:** 10.1186/1471-2164-11-359

**Published:** 2010-06-05

**Authors:** Raoul Frijters, Wilco Fleuren, Erik JM Toonen, Jan P Tuckermann, Holger M Reichardt, Hans van der Maaden, Andrea van Elsas, Marie-Jose van Lierop, Wim Dokter, Jacob de Vlieg, Wynand Alkema

**Affiliations:** 1 Computational Drug Discovery (CDD), Nijmegen Centre for Molecular Life Sciences (NCMLS), Radboud University Nijmegen Medical Centre, Geert Grooteplein Zuid 26-28, 6525 GA Nijmegen, the Netherlands; 2 Department of Molecular Design & Informatics, Schering-Plough, Molenstraat 110, 5342 CC Oss, the Netherlands; 3 Department of Immunotherapeutics, Schering-Plough, Molenstraat 110, 5342 CC Oss, the Netherlands; 4 Molecular Pharmacology Department, Schering-Plough, Molenstraat 110, 5342 CC Oss, the Netherlands; 5 Tuckermann Lab, Leibniz Institute for Age Research - Fritz Lipmann Institute, Beutenbergstraße 11, 07745 Jena, Germany; 6 Department of Cellular and Molecular Immunology, University of Göttingen Medical School, Humboldtallee 34, 37073 Göttingen, Germany

## Abstract

**Background:**

Glucocorticoids (GCs) control expression of a large number of genes via binding to the GC receptor (GR). Transcription may be regulated either by binding of the GR dimer to DNA regulatory elements or by protein-protein interactions of GR monomers with other transcription factors. Although the type of regulation for a number of individual target genes is known, the relative contribution of both mechanisms to the regulation of the entire transcriptional program remains elusive. To study the importance of GR dimerization in the regulation of gene expression, we performed gene expression profiling of livers of prednisolone-treated wild type (WT) and mice that have lost the ability to form GR dimers (GR^dim^).

**Results:**

The GR target genes identified in WT mice were predominantly related to glucose metabolism, the cell cycle, apoptosis and inflammation. In GR^dim ^mice, the level of prednisolone-induced gene expression was significantly reduced compared to WT, but not completely absent. Interestingly, for a set of genes, involved in cell cycle and apoptosis processes and strongly related to Foxo3a and p53, induction by prednisolone was completely abolished in GR^dim ^mice. In contrast, glucose metabolism-related genes were still modestly upregulated in GR^dim ^mice upon prednisolone treatment. Finally, we identified several novel GC-inducible genes from which Fam107a, a putative histone acetyltransferase complex interacting protein, was most strongly dependent on GR dimerization.

**Conclusions:**

This study on prednisolone-induced effects in livers of WT and GR^dim ^mice identified a number of interesting candidate genes and pathways regulated by GR dimers and sheds new light onto the complex transcriptional regulation of liver function by GCs.

## Background

Naturally occurring glucocorticoids (GCs), such as cortisol, play an important role in the regulation of cardiovascular, metabolic and immunological processes. GCs are potent suppressors of inflammatory indices and are widely used to treat chronic inflammatory diseases such as rheumatoid arthritis and asthma [1-3]. However, chronic use of GCs induces side effects, such as diabetes mellitus, fat redistribution, osteoporosis, muscle atrophy, glaucoma and skin thinning [4].

One of the aspects influencing the balance between the desired anti-inflammatory effects and the side effects of GCs is the activation and repression of gene expression. After binding of GCs to the cytosolic GC receptor (GR), the receptor-ligand complex translocates to the nucleus where it alters gene expression. Ligand-bound GR can influence the expression of target genes, either by binding as a dimer to palindromic GC response elements (GRE) or tethering to other DNA-bound transcription factors [5,6].

It is generally assumed that the anti-inflammatory actions of GCs are mainly driven by transrepression, in which ligand-bound GR binds to the pro-inflammatory transcription factors NF-κB (Nuclear factor kappa-light-chain-enhancer of activated B cells), AP-1 (Activator protein 1), IRF-3 (Interferon regulatory factor 3) or other factors, forming an inactive transcription machinery complex, which prevents expression of pro-inflammatory genes [7-16]. However, recent studies demonstrated that under some inflammatory conditions DNA dimer-dependent gene expression could also contribute to the anti-inflammatory activities of the GR [17]. The occurrence of side effects has been linked mainly to transactivation of gene transcription. The well-documented example of GC-induced upregulation of *Pck1 *(*Phosphoenolpyruvate carboxykinase 1*; also known as *Pepck*) and *G6pc *(*Glucose-6-phosphatase*), two genes encoding key enzymes that control gluconeogenesis, is one example linking transactivation to metabolic side effects [18-20].

One of the mechanisms by which GR activates gene transcription is by binding as a homodimer to a GRE in the promoter region of a target gene. GR dimerization involves the D loop located in the DNA-binding domain of the GR, in which several amino acids interact to facilitate receptor dimer formation. It was shown that an A458T point mutation, introduced in the D loop of the GR (GR^dim^), impairs homodimerization and ablates DNA binding [21]. Mice carrying this GR^dim ^mutation are almost as effective as wild type (WT) mice in repressing AP-1 and NF-κB-modulated gene transcription, whereas GR-mediated *Tat *(*Tyrosine aminotransferase*) mRNA induction is largely abolished [21,22].

The mechanism of dimer-dependent transactivation by the GR has been studied in detail for a limited number of GR target genes, including *Fkbp5 *(*FK506 binding protein 5*), *Tat*, *Pck1 *and *Dusp1 *(*Dual specificity phosphatase 1*) [23-26], and tethering-facilitated transrepression of *Mmp1*/*Mmp13 *(*Matrix metallopeptidase 1/13*), *IL-8 *(*Interleukin 8*) and others [27-29]. In order to perform a comprehensive study of the genes and cellular processes that are affected by GC-treatment and influenced by GR dimerization, we have performed genome wide gene expression profiling in liver of prednisolone-treated WT and GR^dim ^mice.

We found that prednisolone predominantly influenced expression of genes involved in glucose metabolism, inflammation, the cell cycle and apoptosis. In general, activation of transcription, including transactivation of known GR marker genes such as *Fkbp5 *and *Tat*, was significantly reduced, but not totally absent in the GR^dim ^mutant. However, a total absence of transcriptional transactivation upon GC-treatment was observed in GR^dim ^mice for a subset of genes, all related to regulation of the cell cycle. In contrast, a small subset of genes was found exclusively regulated by GCs in GR^dim ^mice. Furthermore, we identified *Fam107a *(*Family with sequence similarity 107, member a*; also known as *Tu3a *and *Drr1*) as a novel GC-inducible gene that completely relies on GR dimerization for transactivation.

## Results

### Clustering of individual samples

**Figure 1:**
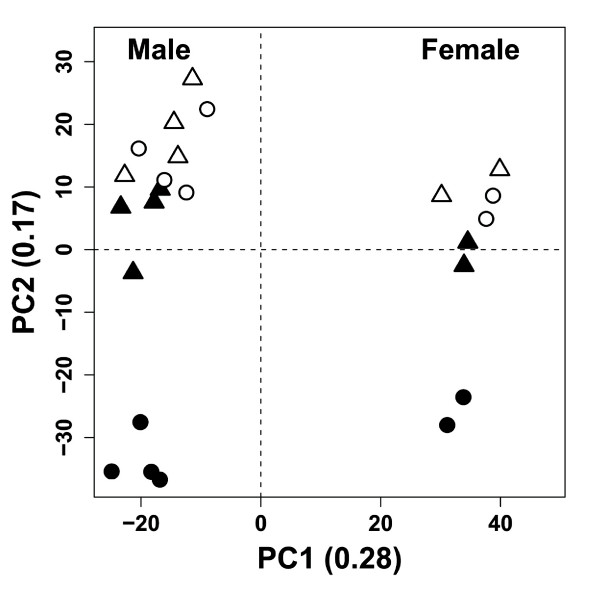
**Principal component analysis on gene expression intensity data**. A principal component analysis (PCA) was performed on normalized gene expression intensity data from 12 wild type (WT) mice (vehicle and prednisolone treatment) and 12 GR^dim ^mice (vehicle and prednisolone treatment). Bullets: WT mice; Triangles: GR^dim ^mice. White symbols: vehicle-treated mice; Black symbols: prednisolone-treated mice. The largest separation was seen between samples derived from male versus female mice (PC1).

**Figure 2:**
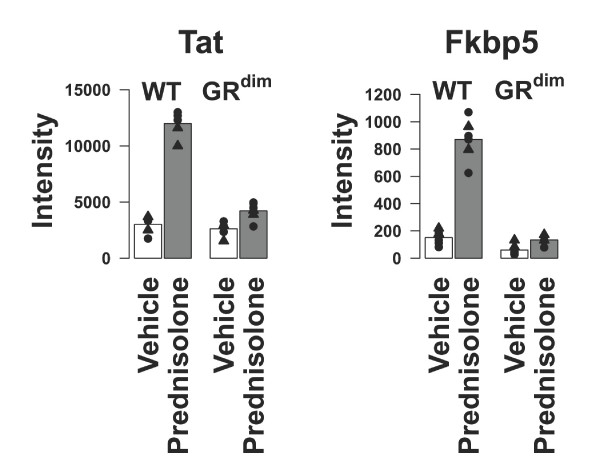
**Intensity profiles of *Tat *and *Fkbp5 *in vehicle and prednisolone-treated wild type versus GR^dim ^mice**. The expression intensities of *Tat *(*Tyrosine aminotransferase*) and *Fkbp5 *(*FK506 binding protein 5*) in vehicle-treated (white bars) and prednisolone-treated (grey bars) wild type (WT) and GR^dim ^mice are plotted. Symbols: Bullets: male mice; Triangles: female mice. Both *Tat *and *Fkbp5 *are known GR marker genes and show strong differential regulation in prednisolone-treated WT mice.

In order to chart the gene expression profile induced by prednisolone in WT and GR^dim ^mice, 6 mice per group were treated with prednisolone or vehicle. A principal component analysis (PCA) on the normalized intensity data showed that the samples clustered into four distinct groups (Figure 1). The largest separation, represented by PC1, was observed between samples derived from male versus female mice. The genes with the highest gender specificity belong to the family of cytochrome P450 proteins, such as *Cyp3a41*, *Cyp3a44 *and *Cyp4a12*, which are known for their gender-specific expression [30,31]. In addition to the gender-based separation, a clear separation was observed between prednisolone-treated and vehicle-treated WT mice, whereas prednisolone-treated GR^dim ^mice clustered closely together with vehicle-treated GR^dim ^and WT mice (PC2). These results indicate that there is a significant prednisolone effect in WT mice, which is strongly reduced in prednisolone-treated GR^dim ^mice. Genes with high loading scores on PC2 include *Tat *and *Fkbp5*, which are known GR-regulated genes in liver [32,33] (Figure 2).

These results show that the transcriptional program that is induced by prednisolone in WT mice is strongly reduced in mice carrying the GR^dim ^mutation. This supports the hypothesis that the loss of GR dimerization leads to a reduction in transcriptional transactivation by the GR and underscores the importance of GR dimerization in induction of gene expression. The effect of prednisolone on up and downregulated genes and pathways will be discussed in more detail below.

### Prednisolone-induced changes in gene expression

To quantify the differences that were seen in the multivariate analysis, differentially expressed probe sets were identified for several treatment comparisons (e.g. prednisolone versus vehicle-treated WT mice and prednisolone vs. vehicle-treated GR^dim ^mice). Probe sets were marked as differentially regulated when the *p*-value, corrected for multiple testing, was below the 0.01 cutoff value.

Prednisolone treatment resulted in a significant differential regulation of 518 probe sets in WT mice (347 upregulated and 171 downregulated; Additional file 1), whereas in prednisolone-treated GR^dim ^mice only 34 probe sets were differentially regulated (29 upregulated and 5 downregulated; Additional file 2). Notably, no differentially regulated probe sets were found in the comparison between vehicle-treated GR^dim ^and vehicle-treated WT mice, suggesting that the GR^dim ^mutation itself does not cause differential gene regulation and that the effects between WT and GR^dim ^mice only become apparent after treatment with prednisolone.

The observation that only 34 probe sets are differentially regulated in GR^dim ^mice upon prednisolone treatment, suggests that there is almost no regulation of gene expression by prednisolone in GR^dim ^mice. However, a large number of the probe sets that are differentially regulated in WT mice showed the same direction of regulation by prednisolone in GR^dim ^mice, but did not meet the cutoff value of 0.01 for the *p*-value. This indicates that induction of gene expression by prednisolone in GR^dim ^mice is strongly reduced but not completely absent (see below).

Taken together these data are in line with the hypothesis that transactivation of gene expression through the GR is significantly reduced in mice carrying the GR^dim ^mutation.

### Biological processes targeted by prednisolone in WT mice

In order to identify cellular processes on which prednisolone has a prominent effect, a gene set enrichment analysis was conducted using the 518 probe sets that were differentially regulated in WT mice by prednisolone. For this analysis we used CoPub, a text mining tool that calculates which biological processes are significantly associated to a set of genes [34]. It appeared that the differentially regulated genes are predominantly involved in three major processes: (glucose) metabolism, cell cycle/apoptosis and immune/inflammatory response (Table 1).

**Table 1:** CoPub keyword enrichment analysis on prednisolone-regulated genes.

Biological process	# of associated genes
**Metabolism**	
Glucose metabolism/transport	22
Gluconeogenesis	21
Lipid metabolism/glycosylation	21
Glycolysis	14
Carbohydrate metabolism/transport	14
Amino acid metabolism/transport	13

**Cell cycle and apoptosis**	
Apoptosis	77
Cell proliferation	72
Cell cycle	65
Cell growth and-or maintenance, cell growth	63
Homeostasis	58
Cell differentiation	49
Cell cycle arrest	37

**Immune/inflammatory response**	
Inflammatory response	31
Acute-phase response	20

CoPub was used to calculate keyword enrichment in the set of prednisolone-regulated probe sets in wild type mice. The analysis revealed that the differential regulated genes (non-redundant set) are predominantly involved in three main processes: metabolism, cell cycle and apoptosis, and immune/inflammatory response. The right column shows the number of differential regulated genes associated with a specific enriched keyword.

**Figure 3:**
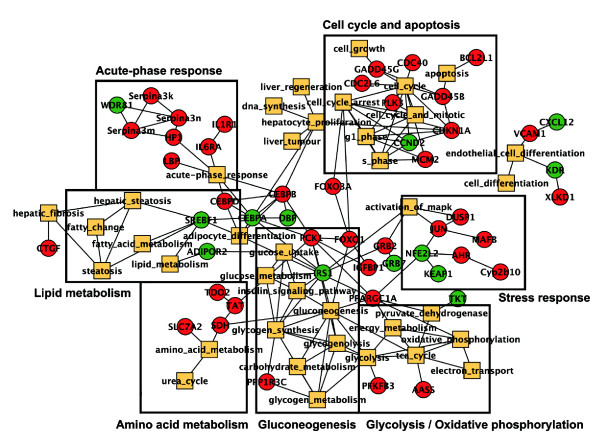
**Literature-based network of glucocorticoid-induced effects**. A network representation of the enrichment results was generated, in which differentially expressed genes together with enriched keywords are plotted. Genes are shown as circles (Red: upregulated; Green: downregulated), whereas enriched keywords are shown as squares. Connections between genes and keywords represent co-publications in Medline abstracts. To avoid an over-complex network, thresholds were set to simplify the interpretation of the results. Only keywords and genes that share at least 5 publications and have an *R*-scaled score of at least 45 are plotted in the network [34]. Several higher-order biological processes that are affected by prednisolone can be appreciated: cell cycle and apoptosis, acute-phase response, stress response, amino acid metabolism, gluconeogenesis and lipid metabolism.

In order to gain insight into the relationships between genes and cellular processes a network representation was created, in which differentially regulated genes are plotted together with enriched keywords. In this representation, cellular processes that are affected by prednisolone can be appreciated as separate areas in the network, such as cell cycle and apoptosis, acute-phase response, and metabolism-related processes such as amino acid metabolism, gluconeogenesis and lipid metabolism. The most influential genes appear as highly connected hubs (Figure 3). Important factors that seem to connect the gluconeogenesis and the cell cycle and apoptosis subnetworks are the transcription factors Foxo1 and Foxo3a (Forkhead box O1 and Forkhead box O3a; see below).

Strong effects of prednisolone treatment on gluconeogenesis, the cell cycle and apoptosis are in line with the anticipated effect of the GR in the liver. The fact that many inflammation and acute phase response-related genes, such as *IL6r *(*Interleukin 6 receptor*) and *Cxcl12 *(*Chemokine (C-X-C motif) ligand 12*) were regulated (Figure 3) is interesting, given that we analyzed livers from healthy mice, not challenged with inflammatory stimuli. The direction and magnitude of prednisolone-induced differential regulation of genes involved in gluconeogenesis and the cell cycle was studied in more detail to get more insight into these prednisolone-affected processes.

### Gluconeogenesis

Table 2 shows the prednisolone-regulated genes in WT mice identified by CoPub as being associated with gluconeogenesis. Among the upregulated genes are *Pck1*, encoding the rate limiting enzyme in gluconeogenesis [35], *Ppargc1a *(*Peroxisome proliferative activated receptor, gamma, coactivator 1 alpha*), a transcriptional coactivator that coordinates the expression of genes involved in gluconeogensis and ketogenesis [36], and *Sds *(*Serine dehydratase*) and *Aass *(*Aminoadipate-semialdehyde synthase*), two genes encoding enzymes involved in amino acid catabolism and amino acid utilization for gluconeogenesis [37,38].

**Table 2:** Prednisolone-regulated genes associated with 'gluconeogenesis' in literature.

Biological process: gluconeogenesis			
Gene name	Symbol	Fc WT	Fc GR^dim^*
*Insulin-like growth factor binding protein 1*	*Igfbp1*	9.7	3.6
*Tyrosine aminotransferase*	*Tat*	4.1	1.6
*6-phosphofructo-2-kinase/fructose-2,6-biphosphatase 3*	*Pfkfb3*	3.9	2.3
*Peroxisome proliferative activated receptor, gamma, coactivator 1 alpha*	*Ppargc1a*	3.2	3.2
*CCAAT/enhancer binding protein (C/EBP), beta*	*Cebpb*	3.1	1.6
*Protein-tyrosine sulfotransferase 2*	*Tpst2*	2.7	1.5
*Solute carrier family 2 (facilitated glucose transporter), member 1*	*Slc2a1*	2.6	1.9
*Forkhead box O1*	*Foxo1*	2.2	1.7
*Serine dehydratase*	*Sds*	2.1	1.5
*Aminoadipate-semialdehyde synthase*	*Aass*	2.1	1.3
*Phosphoenolpyruvate carboxykinase 1, cytosolic*	*Pck1*	1.9	1.3
*Tryptophan 2,3-dioxygenase*	*Tdo2*	1.5	1.2
*Aconitase 2, mitochondrial*	*Aco2*	1.4	1.3
*Transketolase*	*Tkt*	-1.2	-1.1
*Protein kinase, AMP-activated, beta 1 non-catalytic subunit*	*Prkab1*	-1.5	1
*Adiponectin receptor 2*	*Adipor2*	-1.6	-1.3
*Purinergic receptor P2Y, G-protein coupled, 5*	*P2ry5*	-1.6	-1.3
*Pyruvate kinase liver and red blood cell*	*Pklr*	-2.1	-1.4
*CCAAT/enhancer binding protein (C/EBP), alpha*	*Cebpa*	-2.3	-1.2
*Sterol regulatory element binding factor 1*	*Srebf1*	-3	-1.6
*Insulin receptor substrate 1*	*Irs1*	-4	-1.5

CoPub identified 21 prednisolone-regulated genes in wild type (WT) mice that are associated in literature with gluconeogenesis. The identified genes share at least 3 publications and have an *R*-scaled score [34] of at least 35 with keyword 'gluconeogenesis' in Medline abstracts. For each gene, the fold change (Fc) in prednisolone versus vehicle-treated WT mice (Fc WT) is given. Additionally, for each gene also the Fc in prednisolone versus vehicle-treated GR^dim ^mice (Fc GR^dim^) is shown. *It should be noted that in GR^dim ^mice these genes did not meet the *p*-value cutoff of 0.01 for significance after correction for multiple testing, and are mentioned to illustrate a small effect of prednisolone on gene expression in GR^dim ^mice.

The set of downregulated genes includes *Irs1 *(*Insulin receptor substrate 1*), a downstream mediator of the growth factor/insulin signaling pathway that negatively regulates gluconeogenesis [39] and *Pklr *(*Pyruvate kinase*), which encodes a glycolysis associated enzyme known to catalyze the production of pyruvate from phosphoenolpyruvate [40].

The direction of regulation and the function of these genes, suggests that in the liver of prednisolone-treated WT, glucose metabolism is balanced towards gluconeogenesis, which is in line with the reported gluconeogenic effect of glucocorticoids [41,42]. The reduced fold changes in GR^dim ^mice compared to WT mice (Table 2) indicate that glucocorticoid-induced gluconeogenesis is reduced in GR^dim ^mice.

### Cell cycle

Table 3 shows the prednisolone-regulated genes in WT mice that CoPub identified as being associated with the cell cycle. Upregulated genes include *Gadd45b*, *Gadd45g *(*Growth arrest and DNA-damage-inducible 45 beta and gamma*), *Cdkn1a *(*Cyclin-dependent kinase inhibitor 1a*; also known as *p21*) and *Plk3 *(*Polo-like kinase 3*), which are stress sensors involved in cell cycle arrest, DNA repair and apoptosis [43-45]. Other upregulated genes are *Bcl2l1 *(also known as *Bcl2-like1 *and *Bcl-xl*), an anti-apoptotic protein that is enhanced by binding to the Gadd45 family [46,47], and *Dusp1*, a p53 target gene involved in cell cycle regulation [48].

**Table 3:** Prednisolone-regulated genes associated with 'cell cycle' in literature.

Biological process: cell cycle			
Gene name	Symbol	Fc WT	Fc GR^dim^*
*Growth arrest and DNA-damage-inducible 45 gamma*	*Gadd45g*	17.8	2.9
*Dual specificity phosphatase 1*	*Dusp1*	8.2	1
*Cyclin-dependent kinase inhibitor 1A (P21)*	*Cdkn1a*	8.1	1
*Growth arrest and DNA-damage-inducible 45 beta*	*Gadd45b*	5.1	1
*Polo-like kinase 3 (Drosophila)*	*Plk3*	4.5	1
*Retinoblastoma binding protein 8*	*Rbbp8*	4.5	1.3
*Bcl2-like 1*	*Bcl2l1*	3	1.1
*Tensin 1*	*Tns1*	2.6	1.2
*Protein tyrosine phosphatase 4a1*	*Ptp4a1*	2.3	1.1
*Forkhead box O1*	*Foxo1*	2.2	1.7
*PAX interacting (with transcription-activation domain) protein 1*	*Paxip1*	1.9	1
*Forkhead box O3a*	*Foxo3a*	1.9	-1.1
*E1A binding protein p300*	*Ep300*	1.8	1
*NIMA (never in mitosis gene a)-related expressed kinase 7*	*Nek7*	1.8	1.1
*Cell division cycle 2-like 6 (CDK8-like)*	*Cdc2l6*	1.8	1.2
*Cell division cycle 40 homolog (yeast)*	*Cdc40*	1.4	1
*Cyclin D2*	*Ccnd2*	-1.5	-1.2
*NIMA (never in mitosis gene a)-related expressed kinase 6*	*Nek6*	-1.7	-1.2
*CDC28 protein kinase regulatory subunit 2*	*Cks2*	-2.3	-1.3
*TSC22 domain family, member 1*	*Tsc22d1*	-4	-2.3

CoPub identified 65 prednisolone-regulated genes in wild type (WT) mice that are associated in literature with the cell cycle (top 20 shown). The identified genes share at least 3 publications and have an *R*-scaled score [34] of at least 35 with keyword 'cell cycle' in Medline abstracts. For each gene, the fold change (Fc) in prednisolone versus vehicle-treated WT mice (Fc WT) is given. Additionally, for each gene also the Fc in prednisolone versus vehicle-treated GR^dim ^mice (Fc GR^dim^) is shown. *It should be noted that in GR^dim ^mice these genes did not meet the *p*-value cutoff of 0.01 for significance after correction for multiple testing, and are mentioned to illustrate a small effect of prednisolone on gene expression in GR^dim ^mice.

Amongst the downregulated genes are *Ccnd2 *(*Cyclin D2*) and *Cks2 *(*Cdc28 protein kinase regulatory subunit 2*), both involved in cell cycle progression [49,50]. Overall, the direction of the prednisolone-induced differential regulation of genes associated with the cell cycle suggests that prednisolone induces a cytostatic response in liver of WT mice.

### Foxo transcription

From the literature-based network, the two transcription factors Foxo1 and Foxo3a appear to be intermediates between the gluconeogenesis and cell cycle and apoptosis subnetworks (Figure 3). Foxo transcription factors are key mediators of cell cycle progression, apoptosis, glucose metabolism, reactive oxygen species detoxification and DNA damage repair [51-54]. Their activity is tightly controlled by the insulin and growth factor-inducible Pi3k/Akt pathway. Akt-mediated phosphorylation of Foxo transcription factors promotes their translocation from the nucleus to the cytosol and thereby their inactivation through binding of 14-3-3 proteins [54-56].

Foxo transcription factors regulate gene expression of enzymes that are important regulators of gluconeogenesis, such as *Pck1 *and *G6p *[57,58]. In addition, they suppress genes that are involved in glycolysis, the pentose shunt and lipogenesis [59]. Analysis of *Foxo1*-overexpressing mice revealed an increased expression of *Igfbp1 *(*Insulin-like growth factor binding protein 1*), *Pck1*, *Tat *and *Tdo *(*Tryptophan 2,3-dioxygenase*) and downregulation of *Srebf1 *(*Sterol regulatory element binding factor 1*) and *Adipor2 *(*Adiponectin receptor 2*) in liver [59]. Interestingly, these genes are similarly regulated in prednisolone-treated WT mice (Figure 3 and Table 2).

Finally, Foxo transcription factors are regulators of cell cycle progression and were shown to induce *Cdkn1a *and *Gadd45b *[60] and to suppress *Ccnd2 *[54]. Also these genes show the same direction of regulation in prednisolone-treated WT mice (Figure 3 and Table 3). These observations suggest that GCs in WT mice induce Foxo transcription factors, which in turn may synergize with the GR to modulate the expression of their respective target genes.

### Prednisolone-induced differential gene expression in WT versus GR^dim ^mice

**Figure 4:**
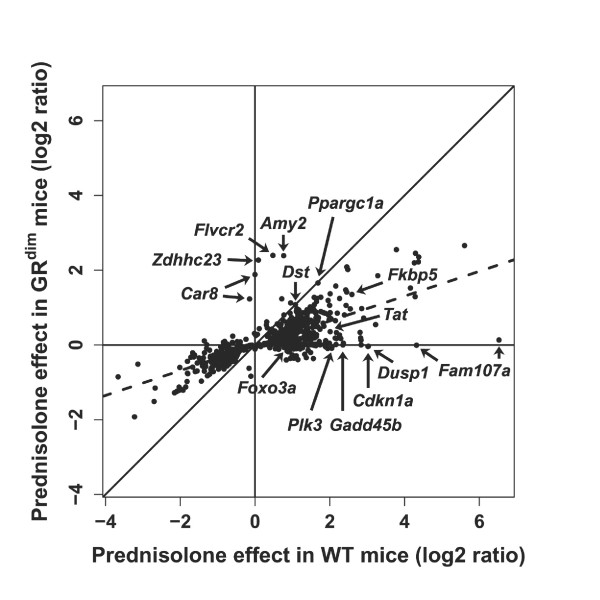
**Log2 ratio plot of prednisolone-induced differential gene expression in wild type versus GR^dim ^mice**. Genes on the solid black line show an equal induction of differential gene expression by prednisolone in wild type (WT) versus GR^dim ^mice. The black-dotted line indicates the average slope of the data and shows that the level of gene regulation in prednisolone-treated GR^dim ^mice is 33% of that observed in WT mice. A subset of genes that show obvious differences in the magnitude of regulation by prednisolone in WT versus GR^dim ^mice is highlighted, as well as genes that show equal magnitude of regulation in WT versus GR^dim ^mice.

Table 2 and 3 list a number of genes that are significantly regulated by prednisolone in WT mice. In the GR^dim ^mice these genes do not meet the significance cutoff for differential regulation (*p *< 0.01), but for most of them a small effect of prednisolone on the expression level can be observed in GR^dim ^mice. To analyze these differences quantitatively, we plotted the log2 ratios of gene expression after prednisolone treatment in WT versus GR^dim ^mice (Figure 4). This figure shows that on average, genes in WT mice are more strongly regulated than in GR^dim ^mice after prednisolone treatment and confirms that induction of gene expression by prednisolone is not abrogated in GR^dim ^mice but reduced to on average 33% of the level in WT mice, as indicated by the slope of the dotted line in Figure 4.

### Identification of genes that differentially respond to prednisolone in GR^dim ^mice as compared to WT mice

In Figure 4, several genes are highlighted that show a different response upon prednisolone treatment in WT mice than in GR^dim ^mice.

Unexpectedly, a small subset of these genes shows stronger upregulation by prednisolone in GR^dim ^mice compared to WT mice. These genes include *Car8 *(*Carbonic anhydrase 8*), a protein associated with proliferation and invasiveness of colon cancer cells [61], *Zdhhc23 *(*Zinc finger, DHHC-type containing 23*), a Nitric oxide synthase-binding and activation protein [62], *Flvcr2 *(*Feline leukemia virus subgroup C cellular receptor family, member 2*), a calcium-chelate transporter [63], and *Amy2 *(*Amylase 2*), an amylase that catalyzes the endohydrolysis of 1,4-alpha-D-glucosidic linkages in oligosaccharides.

**Figure 5:**
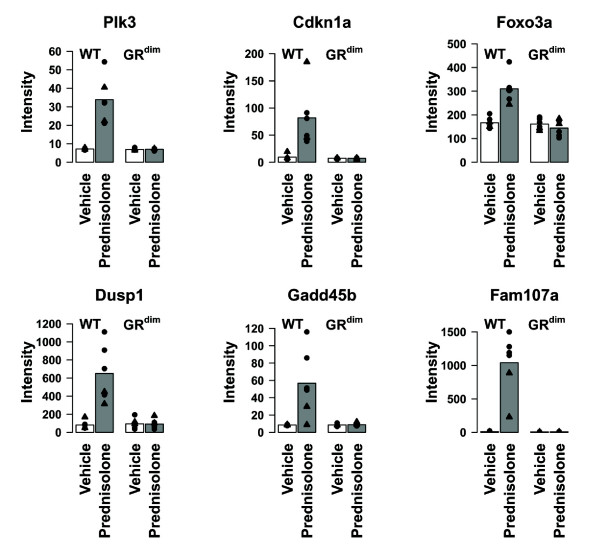
**Intensity profiles of *Plk3*, *Dusp1*, *Gadd45b*, *Cdkn1a*, *Foxo3a *and *Fam107a *in vehicle and prednisolone-treated wild type and GR^dim ^mice**. Several genes were identified that showed differences between prednisolone-treatment in wild type (WT) and GR^dim ^mice. Amongst those genes are *Plk3*, *Dusp1*, *Gadd45b*, *Cdkn1a*, *Foxo3a *and *Fam107a *were differential regulated in prednisolone-treated WT mice, but not in GR^dim ^mice. White bars represent vehicle-treated mice, whereas grey bars represent prednisolone-treated mice. Symbols; Triangles: female mice, Bullets: male mice.

**Figure 6:**
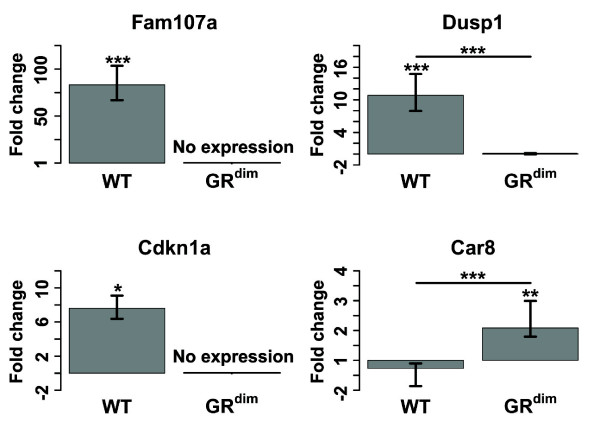
**Validation of microarray-obtained gene expression by Q-PCR**. The relative expression of *Fam107a*, *Dusp1*, *Cdkn1a *and *Car8 *in prednisolone-treated versus vehicle-treated wild type (WT) and GR^dim ^mice was validated by Q-PCR. Differences in expression between two samples were calculated by the 2^ΔΔCt ^method [103,104]. Mean differences in gene expression between WT and GR^dim ^mice were analyzed using the Student's t-test. No expression was observed for *Fam107a *and *Cdkn1a *in vehicle and prednisolone-treated GR^dim ^mice (Ct > 30). Asterisks denote *p*-values as follows: *p < 0.05, **p < 0.01 and ***p < 0.001.

Genes that appear to strictly rely on GR dimerization include *Dusp1*, *Gadd45b*, *Cdkn1a*, *Foxo3a*, *Plk3 *and *Fam107a *(Figure 4 and 5). As discussed above, *Foxo3a*, *Dusp1*, *Gadd45b*, *Cdkn1a *and *Plk3 *all participate in cell cycle regulation, apoptosis and DNA damage repair [43-45,52]. The most striking difference in the level of regulation by prednisolone between WT and GR^dim ^mice was observed for *Fam107a *(also known as *Tu3a *and *Drr1*) (Figure 4, 5 and 6). The expression profile suggests that transcription of *Fam107a *is under direct control of the GR and strictly depends on its dimerization. *Fam107a *encodes a ubiquitously expressed protein that was first described in the context of renal cell carcinoma, in which *Fam107a *expression is reduced or absent due to promotor hypermethylation [64-66]. Overexpression of *Fam107a *in *Fam107a*-negative cell lines leads to growth retardation and apoptosis, indicating that Fam107a might act as a tumor suppressor [65,67]

We also identified a subset of genes that showed an equal induction of gene expression by prednisolone in WT and GR^dim ^mice (located on the diagonal black line in Figure 4); *Dst *(*Dystonin*), a cytoskeletan-interacting protein postulated to cross-link cytoskeletal filaments and thereby maintain cellular integrity [68], *Gtf2a2 *(*General transcription factor II A, 2*), a subunit of the transcription initiation factor TFIIA [69], *Agpat6 *(*1-acylglycerol-3-phosphate O-acyltransferase 6*), an enzyme involved in triglyceride synthesis [70], *Marcks *(*Myristoylated alanine rich protein kinase C substrate*), cytoskeletal protein involved in cell adhesion and cell motility [71], *Obfc2a *(*Oligonucleotide/oligosaccharide-binding fold containing 2A*), a single stranded nucleic-acid-binding protein [72], and *Ppargc1a*.

Prednisolone-induced changes in gene expression in WT and GR^dim ^mice were validated by Q-PCR for *Fam107a*, *Dusp1*, *Cdkn1a *and *Car8*. Figure 6 shows the fold-changes of the selected genes in prednisolone versus vehicle-treated WT and GR^dim ^mice. For all four genes, the regulation by prednisolone in WT and GR^dim ^mice was qualitatively similar to what was found by microarray analysis.

## Discussion and Conclusion

In this study we performed liver gene expression profiling of WT and GR^dim ^mice after prednisolone administration. Our aim was to chart the biological processes in the liver that are affected by GCs and to study their dependence on DNA-binding and dimerization of the GR. CoPub keyword enrichment analysis with prednisolone-regulated genes in WT mice showed enrichment of keywords associated with glucose, lipid and amino acid metabolism, the cell cycle and apoptosis (Table 1 and Figure 3). This is in agreement with cellular processes known to be affected by GCs [73,74]. Interestingly, the forkhead transcription factors Foxo1 and Foxo3a are regulators of these cellular processes and induced by prednisolone in WT mice. Together with the observation that a subset of the prednisolone-induced genes are Foxo1 and Foxo3a target genes and are similarly regulated in Foxo1 and Foxo3a expression profiling experiments [54,59,60], this suggests that the GR synergizes with these transcription factors in mouse liver to control lipid and glucose metabolism and the cell cycle. This concept is further supported by the recent finding that the *MurF1 *(*Muscle RING finger 1*) promoter contains adjacent binding sites for the GR and Foxo transcription factors and is synergistically activated when both are co-expressed [75]. However, it is likely that a subset of the prednisolone-induced genes is under direct control of Foxo1 and Foxo3a without the need for synergy with the GR. Other studies, addressing small gene sets or individual genes, had also identified a role for Foxo1 and Foxo3a in GR-induced gene expression [58,75-77].

The list of differentially regulated genes by prednisolone in WT mice overlaps with that of an earlier study by Phuc Le *et al*, which combined chromatin immunoprecipitation (ChIP) data with gene expression data to identify direct GR target genes in mouse liver [78]. Most of the genes found in our study displayed the same up and downregulation of gene expression, such as *Tat*, *Foxo1 *and *Fkbp5 *that are upregulated and *Adipor2, CCAAT/enhancer binding protein alpha *(*C/EBPα*) and *Tkt *that are downregulated. From a candidate gene set of 302 genes, Phuc Le *et al *identified metabolism, cell proliferation and programmed cell death as important processes in their GO-term analysis, which is in agreement with our findings.

Interestingly, in contrast to our study, Phuc Le *et al *did not observe upregulation of bona fide GR target genes, such as *Pck *and *Igfpb *in fed CD1 mice and explain this by lack of response due to a potential inhibition by insulin signaling in the fed state [79]. The fact that we observed upregulation of these genes indicates that in our experimental setup, which differs in several aspects from the setup used by Phuc Le *et al*, such as mouse strain used for the study, GC dosage and the fed state of the mice, the inhibitory effects of fed-induced insulin signaling do not play a role.

The overall induction of gene expression was strongly reduced in prednisolone-treated GR^dim ^mice compared to WT mice (Figure 4). Nevertheless, in many cases residual gene induction by prednisolone was still observed. This indicates that GR dimerization is indeed an important mechanism for activation of these genes, although some regulation can take place even in the absence of GR homodimers. In fact, GR monomers are in principle capable of binding to GC response elements (GREs) and evoking a basal induction of gene expression. However, due to the lack of cooperative binding they are less potent than GR homodimers. This is in line with the regulation reported for *Pnmt *(Phenylethanolamine-N-methyltransferase) and *Amy2 *in which binding of GR monomers to GREs or a GRE half-site (i.e. only one-half of the classical palindrome) was sufficient to confer induction by GCs [80,81]. In case of the *Pnmt *gene, multiple GRE half-site have been identified in the promoter region allowing receptor clustering and thereby stable binding of GR monomers independent of the DNA-binding domain (DBD) interface [80]. This also explains why expression of the *Pnmt *gene is not compromised in GR^dim ^mice despite the lack of GR dimer formation [21]. Another explanation for the residual gene induction by prednisolone in GR^dim ^mice is that the mutant GR still forms homodimers but that these are far less stable than in WT mice.

A recent study showed that the specific sequence of a GRE differently affects the conformation of the GR and thereby its activity towards specific target genes [82]. Mutation analysis of overexpressed GR domains that are involved in transcriptional activation, namely the dimerization region in the DBD (Dim) as well as AF1 and AF2 (activation function 1 and 2), showed that the dependency on each of them was specific for the sequence of the GR binding site and that genes differed in their dependence on dimerization [82]. The fold induction of *Tat *and *Fkbp5 *by dexamethasone in the Dim mutant was around 30% for *Tat *and 50% for *Fkbp5 *of that for WT GR, which is in gross agreement with our own observations *in vivo *(Figure 4).

The effect of the GR^dim ^mutation was found in all major pathways that were induced by prednisolone, i.e. for genes in inflammatory pathways, gluconeogenesis and cell cycle. Moreover, the attenuating effect of the GR^dim ^mutation was found for genes that were upregulated as well as for genes that were downregulated by prednisolone. This suggests that genes, which fail to be repressed by prednisolone in GR^dim ^mice, are either regulated through GR binding to negative GRE elements (nGREs) or indirectly regulated via other GR target genes. Interestingly, analysis of the magnitude of gene regulation by prednisolone in WT mice compared to GR^dim ^mice showed that the cell cycle-related genes are more dependent on the dim interface than genes related to gluconeogenesis (Table 2 and 3). This can also be appreciated in Figure 4; several genes that are on the x-axis (i.e. show no differential gene expression upon prednisolone treatment in GR^dim ^mice), are cell cycle-related.

We identified several genes that showed strong upregulation by prednisolone in GR^dim ^mice compared to WT mice, such as *Amy2*, *Car8 *and *Zdhhc23*, and several genes that showed equal induction of gene expression by prednisolone in WT and GR^dim ^mice, amongst them are *Dst *and *Ppargc1a *(Figure 4). These genes are potential candidates for having GRE half-sites in their promotor regions, which could explain why these genes show equal or even higher induction of gene expression by prednisolone compared to WT mice. As mentioned earlier, the presence of GR half-sites in the promotor region for *Amy2 *was indeed experimentally confirmed [81]. For the other genes however, we did not find literature evidence for the presence of GR half-sites in their promotor regions. Therefore, follow-up studies on these genes to determine the functional GRE sites in their regulatory region would be of interest to study the significance of dimer-interface independent GRE binding. Genes that showed equal or stronger upregulation by prednisolone in GR^dim ^mice compared to WT mice can also be secondary response genes; under transcriptional control of transcription factors other than the GR. Keyword enrichment analysis performed with this set of genes did not identify enrichment for a particular cellular process.

We identified several genes that are absolutely dependent on GR dimerization for the induction of gene expression by prednisolone, such as *Cdkn1a*, *Gadd45b*, *Dusp1*, *Plk3 *and *Foxo3a *(Figure 4). Interestingly, all of these have a strong relationship with p53: some are under direct transcriptional control of p53 (*Cdkn1a *[83], *Gadd45b *[84] and *Dusp1 *[48]) or physically interact with p53 (Foxo3a [85,86] and Plk3 [87]). The cellular responses mediated by Foxo3a and p53 are highly similar, share some of their target genes (e.g. *Cdkn1a *and *Gadd45b*) and use similar mechanisms to regulate post-translational modification [51,88]. Therefore, Foxo3a and p53 can be regarded as partners that positively as well as negatively regulate each other, depending on the context [51]. This observation might indicate that GC-induced activation of Foxo3a and/or p53 is hampered in GR^dim ^mice. In line with this hypothesis Foxo3a was recently shown to be required for GC-induced apoptosis in lymphocytes [89]. Interestingly, this process is defective in GR^dim ^mice highlighting a possible link between the GR, Foxo3a and induction of lymphocyte apoptosis [21].

*Fam107a *showed the largest induction of gene expression by prednisolone in WT mice (Figure 4 and 5). Analysis of protein-protein interactions revealed that Fam107a interacts with Tada2a [90,91], a protein that together with binding partner Tada3a (Transcriptional adaptor 2 and 3 alpha) are core proteins of the histone acetyltransferase (HAT) complex [92]. HAT complexes are involved in chromatin structure modification for initiation of gene transcription, but can also acetylate non-histone proteins to modify their activity and stability [92,93]. Interestingly, Fam107b a paralog of Fam107a with 84% protein similarity (not regulated in mouse liver by prednisolone) was shown to interact with Tada3a [94].

The observation that Fam107a inhibits cell proliferation and induces apoptosis when overexpressed [65,67], suggests that Fam107a, like Foxo3a, Dusp1, Gadd45b, Cdkn1a and Plk3 play a role in regulating the cell cycle. Furthermore, the association of Fam107a with a core protein of the HAT complex might indicate that Fam107a may serve as a cofactor in the transcription machinery complex.

The activity and function of Foxo3a and p53 are strongly modulated by acetylation [51,88,95]. Hence, *Fam107a *is an interesting candidate gene for follow up experiments to study whether it modulates GR-induced gene expression and/or acetylation of GR-associated transcription factors such as Foxo3a, p53, C/EBPα and C/EBPβ (CCAAT/enhancer binding protein, beta).

For a more comprehensive view on GC regulated process in the liver, experiments with multiple time points, different doses of GCs and using one or more inflammatory stimuli, could be considered.

## Methods

### Animals

All mice (WT and GR^dim^; Balb/c) were bred at the Institute of Virology and Immunobiology at the University of Würzburg. In total 24 mice (8 male WT, 4 female WT, 8 male GR^dim ^and 4 female GR^dim^) were included in the study. Mice were treated subcutaneously with 1 mg/kg prednisolone (5% DMSO and 5% Chremophor in manitol, 10 ml/kg) once and sacrificed 150 minutes later by cervical dislocation, which was approved by the responsible authorities in Bavaria (Regierung von Unterfranken). All experiments were performed in the morning between 9 and 10 AM. The mice were exposed to a regular dark-light-cycle (lights on between 6 AM and 6 PM) and had access to water and food ad libitum at any time.

### RNA isolation

Liver biopsy specimens were collected into aluminum containers, snap freezed in liqN_2 _and stored at -80°C before use. RNA isolation was done using Trizol, followed by RNeasy clean-up to enhance the A_260_/A_230 _ratio. RNA quantity and quality was determined using the NanoDrop Spectrophotometer and Agilent Bioanalyzer. For all samples subjected to microarray hybridization, the RIN (RNA integrity number) was 9.0 - 10.

### Microarray data processing

Processed RNA samples were hybridized on GeneChip Mouse Genome 430 2.0 arrays (MOE430-2) from Affymetrix [96]. Processing and downstream statistical analysis of the microarray data was done using the *R *Statistics package [97]. Data were normalized using the gcrma algorithm, pair-wise ratios between treatments were built using the limma package and annotation for the probe sets was derived from the mouse4302 library, all as provided in BioConductor [98]. In all contrast matrices, a correction for gender type was applied. Data were deposited in the NCBI Gene Expression Omnibus (GEO), accession number GSE21048.

### Keyword enrichment analysis

Keyword enrichment analysis on the microarray data was performed using CoPub [34] with default settings as provided by the web server [99]. The literature-network between enriched keywords and genes (nodes) and their co-publications (edges) were visualized using Cytoscape software [100,101].

### Validation of microarray results with Q-PCR

Total RNA (1 μg) was reverse transcribed using a commercially available cDNA synthesis kit (iScript, BioRad Laboratories, Hercules, CA, USA). Q-PCR was performed by SYBR Green-based quantification according to the manufacturer's protocol (Applied Biosystems, Foster City, CA, USA). Primers were developed for Fam107a (Family with sequence similarity 107, member a; Fw:TCATCAAACCCAAGAAGCTG; Rev: CTCAGGCTTGCTGTCCATAC), Car8 (Carbonic anhydrase 8; Fw: CACACCATTCAAGTCATCCTG; Rev: ACCACGCTGGTTTTCTCTTC), Cdkn1a (Cyclin-dependent kinase inhibitor 1a; Fw: TCTTGCACTCTGGTGTCTGAG; Rev: ATCTGCGCTTGGAGTGATAG) and Dusp1 (Dual specificity phosphatase 1; Fw: GTGCCTGACAGTGCAGAATC; Rev: CCAGGTACAGGAAGGACAGG) using Primer3 [102]. All primer pairs were exon-spanning. The gene Azgp1 (alpha-2-glycoprotein 1, zinc-binding; Fw: AAGGAAAGCCAGCTTCAGAG; Rev: ACCAAACATTCCCTGAAAGG) was chosen as endogenous control since the expression arrays did not show any differences in expression of this gene between the two experimental groups (WT vs GR^dim^). PCR products were selected to be between 80 and 120 bp long. Samples were run on the 7500 Fast Real-Time PCR System (Applied Biosystems) using the following protocol: 10 min. denaturation at 95°C, and 40 cycles of 15 sec. denaturation at 95°C, 60 sec. annealing and extension at 60°C. All primer pairs were validated in triplicate using serial cDNA dilutions. Primer pairs that were 100 ± 10% efficient, which implies a doubling of PCR product in each cycle, were used to quantify mRNA levels. Threshold cycle numbers (referred to as Ct) were obtained using the 7900 HT System SDS software version 2.3 (Applied Biosystems). All samples were measured for three times and samples with a standard deviation (SD) larger than 0.5 were excluded from the analysis. The relative quantity (RQ) of the gene-specific mRNA was calculated from the average value of the ΔCt (target gene Ct - endogenous control gene Ct) for each of the 24 analyzed samples. Differences in expression between two samples were calculated by the 2^ΔΔCt ^method [103,104]. For Q-PCR, mean differences in expression between groups were analyzed using the Student's t-test. A *p*-value of < 0.05 was considered statistically significant in each situation.

### Protein - protein interaction data

Protein-protein interaction data were retrieved from the Biological General Repository for Interaction Datasets (BioGRID) database [105,106].

## Abbreviations

GR: Glucocorticoid receptor; WT: Wild type; GR^dim^: GR with the A458T point mutation in the dimerization region of the DNA-binding domain; GCs: Glucocorticoids; GRE: Glucocorticoid response element.

## Authors' contributions

RF, AvE, MJvL, JPT, HMR, WD, JdV and WA designed research. JPT and HMR generated and provided GR^dim ^and WT mice. HvdM performed RNA isolations and microarray hybridization experiments. EJMT designed and performed Q-PCR experiments. RF, WF and WA analyzed the microarray data and wrote the paper. All authors read and approved the final manuscript.

## Supplementary Material

Additional file 1 **Differentially regulated probe sets in liver of prednisolone-treated versus vehicle-treated wild type mice**. Prednisolone treatment resulted in a significant differential regulation of 518 probe sets in wild type mice (347 upregulated and 171 downregulated; p-value < 0.01). The table shows the Affymetrix probe set identifiers of the differentially regulated probe sets, full gene names, gene symbols and the fold changes. Click here for file

Additional file 2 **Differentially regulated probe sets in liver of prednisolone-treated versus vehicle-treated GR^dim^mice**. Prednisolone treatment resulted in a significant differential regulation of 34 probe sets in GR^dim ^mice (29 upregulated and 5 downregulated; p-value < 0.01). The table shows the Affymetrix probe set identifiers of the differentially regulated probe sets, full gene names, gene symbols and the fold changes. Click here for file
